# The role of dual mobility total hip arthroplasty to reduce risk of dislocation in patients with neurological disorders. a systematic review

**DOI:** 10.1007/s00590-025-04476-1

**Published:** 2025-08-29

**Authors:** Francesco Cavola, Daniele Grassa, Alessandro Singlitico, Guido Bocchino, Giulio Maccauro, Raffaele Vitiello

**Affiliations:** Ortopedia e Traumatologia, Università Cattolica del Sacro Cuore, Roma, Rome, Italy

**Keywords:** Femoral neck fractures (FNF), Total hip arthroplasty (THA), Dislocation rate, Parkinson’s disease (PD), Dual mobility implants

## Abstract

**Background:**

Femoral neck fractures (FNF) are a significant cause of morbidity and mortality in older adults, often requiring total hip arthroplasty (THA). However, THA is associated with high dislocation rates, particularly in patients with neurological disorders due to factors such as muscle weakness, cognitive impairments, and frequent falls. Dual mobility cups (DMCs) have been proposed as a solution to enhance stability and reduce dislocation rates in this high-risk population. This systematic review aims to evaluate the effectiveness of DMCs in reducing dislocation rates and improving clinical outcomes in patients with FNF and neurological disorders undergoing THA.

**Methods:**

Following PRISMA guidelines, a systematic search was conducted across PubMed, Cochrane, Embace and Google Scholar up to June 2024. Studies included randomized controlled trials, clinical trials, and retrospective studies focused on DMC use in THA for patients with neurological conditions. Outcomes analyzed included dislocation rates, complications, and patient-reported outcomes such as mobility levels.

**Results:**

Data from 12 studies (2017–2023) involving 588 patients were included. The overall mean age of participants was 76.85 years, with 229 males and 376 females. Neurological conditions included Parkinson’s disease, dementia, stroke, and others. Dislocation rates were significantly low, with only 2 dislocations reported (0.34%), compared to historically higher rates in standard THA. Complication rates were modest (6%) and primarily involved periprosthetic fractures and infections. Functional outcomes, assessed using tools like the Harris Hip Score (HHS) and WOMAC, demonstrated significant postoperative improvement, with most patients achieving “good” to “excellent” outcomes.

**Conclusion:**

Dual mobility cups in THA significantly reduce dislocation rates and provide favorable functional outcomes in patients with FNF and neurological disorders. DMCs appear to be a safer alternative for high-risk populations, offering improved stability and quality of life. Future studies should focus on long-term outcomes, including implant durability and revision rates.

## Introduction

Parkinson’s disease (PD) and other neurological disorders have long been linked to specific challenges in patients undergoing total hip arthroplasty (THA) [1] Increased muscle tone and impaired motor control contribute to complexities in both intraoperative and postoperative management of these patients. Evidence shows that PD is associated with higher complication rates in THA patients, particularly regarding joint instability and issues related to postoperative rehabilitation [2]. Additionally, it is well-established that PD patients may experience general functional decline following surgery due to the progressive nature of the disease [3] There is also concern about an elevated risk of postoperative cognitive decline in PD patients [4], as cognitive impairment may further increase dislocation risk by reducing adherence to postoperative restrictions.

Hip Osteoarthritis in elderly people and Femoral neck fractures (FNF) pose significant challenges for orthopedic surgeons due to the presence of severe comorbidities that affect pre and postoperative course of patients; in particular Femoral neck fractures are a major source of mortality and disability among older adults [5–7]. Many patients with FNF meet criteria for total hip arthroplasty (THA). However, the dislocation rate for THA in these patients is approximately 20%, and recurrent early dislocations can lead to revision surgeries and a high rate of associated complications [8]. Factors contributing to post-THA instability include both patient-related factors (such as gender, age, and abductor muscle weakness) and surgical factors (such as the surgical approach, component positioning, femoral head size, and the range of motion) [9, 10]. Patients with FNF are particularly susceptible to prosthetic dislocation compared to those with hip arthritis, due to factors like: muscle weakness, cognitive and neurological impairments, and frequent falls that are common in this patients’ population [11]. In cases of recurrent instability, salvage procedures such as converting to Dual Mobility implant or adding a constrained liner have been used, though they often reduce functional outcomes and implant durability [12].

Recently, dual mobility acetabular components have gained attention as a potential solution for preventing and managing instability in both primary and revision THA procedures [13]. The dual mobility design retains the smaller head size, minimizing wear, while adding an ultra-high-molecular-weight polyethylene (UHMWPE) insert that acts as a larger femoral head, providing a broader range of motion. This dual articulation system allows the femoral head to move against the inner surface, while the outer surface moves against the metal shell, enhancing stability without compromising clinical outcomes or implant longevity [14, 15].

We have found a lack of recent evidence concerning total hip arthroplasty (THA) in patients with Parkinson’s disease (PD) and other neurological disorders, especially regarding published outcome studies for the newer dual mobility implants currently available, which allow better bone fit and fixation, use innovative materials and are always closer to a per patient personalized implant [16].

In this study, our primary goal is to evaluate how effective dual mobility cups are to prevent dislocation in people affected by neurological conditions, who undergo this type of surgery due to femoral neck fractures and osteoarthritis. Secondary, we will also investigate patient-reported outcomes, through functional scores, used in the studies we included in this review, such as: Harris Hip Score (HHS), UCLA Activity Scale and Western Ontario and McMaster Universities Osteoarthritis Index (WOMAC).

## Materials and methods

The review adhered to the PRISMA (Preferred Reporting Items for Systematic Reviews and Meta-Analyses) guidelines [14], ensuring a comprehensive and systematic approach to data retrieval and synthesis. The study has been registered in PROSPERO and the protocol number is CRD420251077317.

### Search strategy

The analysis was conducted using the keywords ‘dual mobility’, ‘bis mobility’, ‘dual-mobility’, ‘bis-mobility’, ‘bismobility’, ‘dualmobility’ and ‘neurological’, ‘neuromuscolar’, ‘parkinson’, ‘dystonia’, ‘parkinsonism’, ‘dementia’, ‘cognitive’, ‘alzheimer’. Databases searched included Medline (PubMED), Cochrane, Embase and Google Scholar up to June 30, 2024. Articles published in English, Spanish, French, Portuguese and Italian in peer-reviewed journals were considered. Excluded were biomechanical reports, animal studies, cadaver studies, in vitro research, case reports, case series with fewer than 10 cases, literature reviews, technical notes, letters to editors and instructional materials. 2 authors (A.S. and G.B.) independently reviewed abstracts and full texts were obtained if abstracts were inconclusive. All differences between the reviewers were discussed and if disagreement remained the senior author (R.V. or D.G.) was consulted. Reference lists of selected articles were manually checked. All the selected studies were retrospectively analyzed by an author (D.G.) who then extracted and entered the data in an Excel worksheet. Lastly, the data sheet was reviewed by 2 authors (F.C. and R.V.) who agreed on the extracted data.

### Inclusion and exclusion criteria

The eligibility criteria for inclusion in our analysis were set to ensure the selection of studies meeting rigorous standards. Studies were included if designed as a randomized controlled trials, clinic trials and Retrospective studies. Exclusion criteria were studies designed as systematic review, meta-analysis, experimental studies (in vitro studies, animal studies or cadaveric studies). The inclusion criteria for the selected articles include: Patients with positive anamnesis for neurodegenerative diseases, diagnosed with medial femoral fractures and hip osteoarthritis, who underwent total hip arthroplasty (THA) with a dual mobility cup. We considered studies written in English, Spanish, French, Portuguese and Italian. Inclusion and exclusion criteria are presented in Table 1. The 3 reviewers (G.B., D.G., F.C.) evaluated the full text of the selected articles to determine whether it was eligible for inclusion and collected data of interest. In case of doubt regarding the inclusion of an article, the senior author made the final decision. The 3 authors (G.B., D.G., F.C.) independently assessed the risk of bias. A supervisor (G.M.) was consulted in case of disagreement.

**Table 1:** Inclusion and exclusion criteria

	**Inclusion**	**Exclusion**
Population	Patients diagnosed with medial femoral fractures and hip osteoarthritis, who underwent total hip arthroplasty (THA) with a dual mobility cup; patients diagnosed with neurodegenerative disease	Pediatric population, open fracture, other fracture associated, soft tissue injuries
Intervention	Dual mobility total hip arthroplasty	Hemiarthroplasty, revision hip implants and standard THA
Design	Randomized controlled trials, clinic trials and retrospective studies	Other designs (e.g. systematic review, opinions commentaries and case report)
Other	English, Spanish, French, Portuguese and Italian	Not in English, Spanish, French, Portuguese and Italian

### Data extraction and analysis

Detailed information was systematically extracted from each selected study. The selected studies covered a range of variables including demographic data, type of diagnosis, surgical methods, and outcomes related to dislocation and return to usual stand life. Descriptive statistics were used to summarize the findings across all the included studies.

## Results

### Search and literature selection

**Fig. 1:**
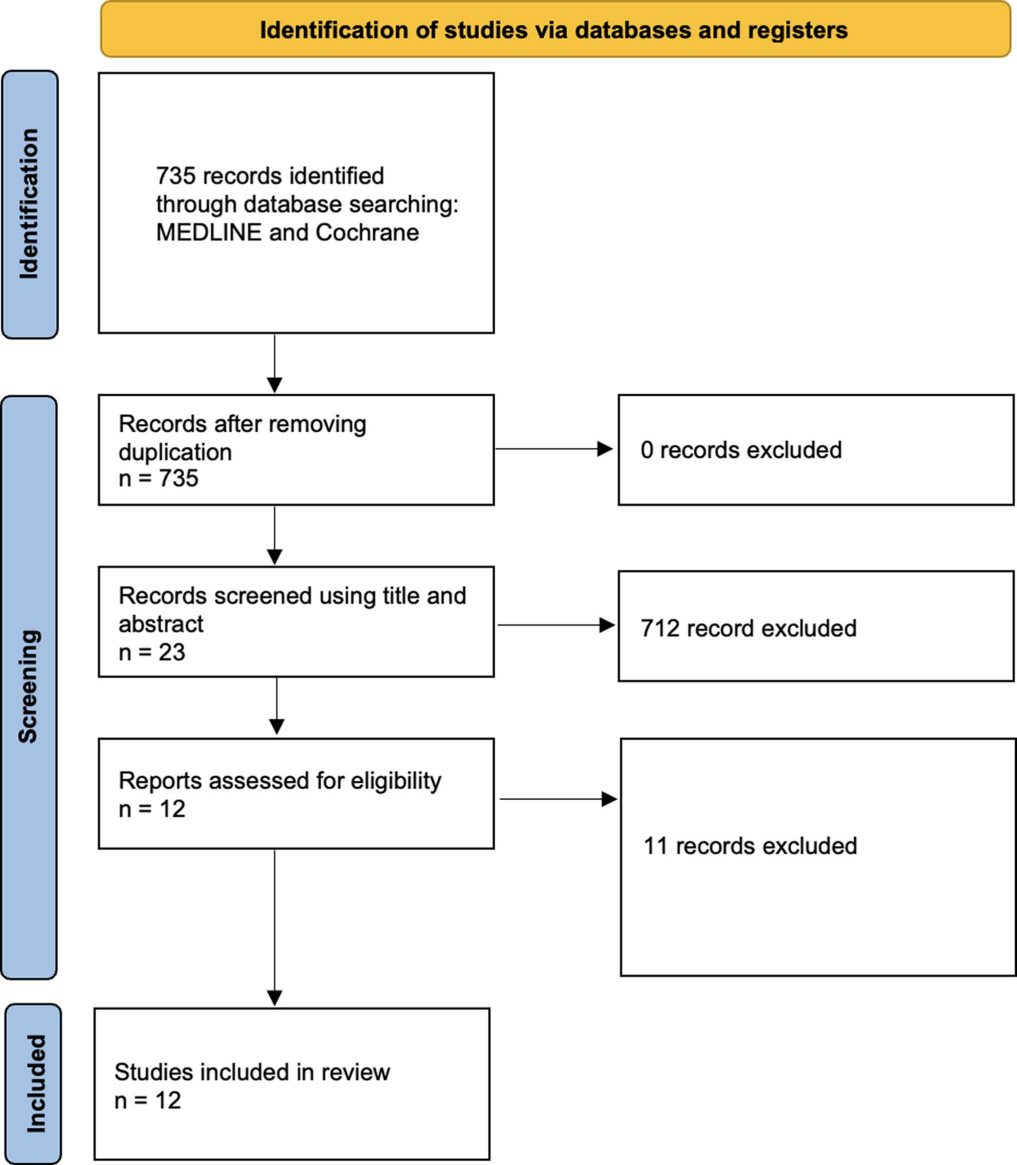
PRISMA flowchart. PRISMA, Preferred Reporting Items for Systematically Reviews and Meta-Analyses

The data analyzed come from scientific studies published between 1993 and 2024. An initial literature search identified 735 papers for potential evaluation. Before starting the screening process, 715 papers were excluded using title and abstract, leaving 20 for further review as they did not meet the inclusion criteria. From the remaining studies, 8 were eliminated based on detailed inclusion and exclusion criteria. In the end, 12 papers fulfilled all the required criteria for inclusion (Fig. 1).

### Demographics

This systematic review analyzes data from 12 studies conducted between 2017 and 2023, encompassing a total of 588 patients diagnosed with neurological disorders who underwent Total Hip Arthroplasty with a dual mobility cup. The included studies represent a range of methodologies, including prospective cohort studies, retrospective studies, randomized controlled trials and clinical trials, offering a comprehensive evaluation of the outcomes associated with this surgical technique. The mean age of participants showed considerable variation across the studies, with an overall average of 76.85 years ( rage 24–90 years). Regarding gender distribution, 229 patients were male, and 376 were female; however, one study did not provide gender-specific data [Table 2].

**Table 2:** Overview of Studies, Patient Characteristics, and Study Designs

				Gender			
Ref	Year	Type of the study	N°of patients	M	F	Mean age (years)	Follow-up
*El-Deeb MA *et al*. *[27]	2023	Prospective cohort study	20	13	7	70.5 ± 6.42	24 months
*Liang C *et al*. *[28]	2023	retrospective study	17	21	37	75.2 ± 9.2	12.3 months
*Ryu HG *et al*. *[29]	2021	Retrospective cohort study	35	8	27	77.6 ± 8.4	> 5 years
*Lazennec JY *et al*. *[3]	2018	retrospective study	42	34	25	72.5 (55–79)	8.3 years
*Ochi H *et al*. *[30]	2017	retrospective study	33	7	26	80 (61–95)	15.8 months
*Harwin SF *et al*. *[31]	2017	retrospective study	249	103	146	66 (24–90)	3,3 years
*Iorio *et al*. *[32]	2019	randomized control trial	30	12	18	82	24 months
*Nonne D *et al*. *[33]	2019	retrospective clinica trial	60	15	45	87,6	28,3 months
*Bassiony *et al*. *[34]	2020	prospective cohort study	13	7	6	65	32 months
*Godoy-Monzon *et al*. *[35]	2020	prospective study	41	N/A	N/A	85.2	28 months
*Alberio R *et al*. *[36]	2021	retrospective clinica trial	28	7	21	77,6 (71–85)	23 months
*Graversen A *et al*. *[18]	2017	retrospective clinica trial	20	2	18	83 (81–88)	12,1 months
***Total***			588	229	376	76.85	

### Type of neurological disease

All patients considered, in the studies we included in this systematic review, were diagnosed with neurological diseases such as: Stroke, Hemiparesis, Alzeheimer, Parkinsonism, Epilepsy, Dementia, Psychic depression, old poliomyelitis, ICHge, Cerebral palsy. Most represented diagnoses were Parkinson disease (more than 95 cases) and Dementia (more than 53 cases). However, 2 studies mentioned that all patients included in the study were affected by neurological diseases without reporting diagnoses and its distribution (Table 3).

**Table 3:** Distribution of Neurological Disorders

Ref	N°of patients	Neurological diseases
*El-Deeb MA *et al*.*[27]	20	Stroke, Hemiparesis (5); Alzeheimer,Parkinsonism (4); Epilepsy (3); Dementia (3); Psychic depression (3); old poliomyelitis (1); Intracerebral Hemorrhage (1)
*Liang C *et al*. *[28]	17	n/a
*Ryu HG *et al*. *[29]	35	Cerebral palsy, poliomyelitis, hemiplegia, paraplegia, and Parkinson disease
*Lazennec JY *et al*.*[3]	42	Parkinson disease
*Ochi H *et al*. *[30]	33	Parkinson disease
*Harwin SF *et al*. *[31]	249	Neurodegenerative disorder
*Iorio *et al*. *[32]	30	Dementia
*Nonne D *et al*. *[33]	60	n/a
*Bassiony *et al*. *[34]	13	Parkinson disease
*Godoy-Monzon D *et al*. *[35]	41	Parkinson disease, Alzheimer disease, neurological deficit, Dementia, abductor system deficiency
*Alberio R *et al*. *[36]	28	Parkinson (3), Hemiplegia (1), hemiparesis (8)
*Graversen A *et al*. *[18]	20	Dementia

### Diagnose, surgical approach and type of implant

All the studies included in the review considered only Femoral neck fracture diagnose, exception made by *Lazennec JY *et al*. *[3] who selected a population of 42 adults affected by Hip Osteoarthritis. Implants chosen for all patients included in the review were Dual Mobility Cups in THA. All three mostly used surgical approaches to the hip were used (Posterolateral, Anterior, Direct Lateral approach). Two of the studies included did not report details about the surgery performed. In 63 cases surgeons preferred Hardinge Direct Lateral Approach. In 90 cases a Smith-Petersen Anterior approach was used and 165 patients were operated with a Posterolateral approach described by Gibson-Moore (Table 3).

### Rate of dislocation

Eleven out twelve studies included in this systematic review reported zero dislocations during the follow-up, just *Ryu HG *et al*.* [29] experienced 2 dislocations out of 35 patients treated (5.7%), not related to periprosthetic fracture or septic mobilization. Overall the total dislocation rate was 0.34% (Table 3).

### Clinical outcomes

Clinical outcomes were not mentioned and considered in all the studies that have been included in this review. Three of them used the Harris Hip Score (HHS) to evaluate the pre and post surgery patient’s performances. *El-Deeb MA *et al*.* [27] have registered a pre-operative HHS range of 15–40 in their patients pool, with an increase after 2 years from the operations till 92–99. Good results were proposed also by *Ryu HG *et al*. *[29] with a final range of 81.5 ± 13.5 and *Harwin SF *et al*. *[31] who reported a mean postoperative HHS of 92.5. *Ryu HG *et al*. *[29] also assessed the patient’s physical activity level using the UCLA Activity Score obtaining an unchanged score (4.6 ± 1.5) in relation with the period foregoing the hip fracture and successive the implant of TKA. Similar results were registered in *Ochi H *et al*. *[30] study were no significant difference between groups regarding pre- and postoperative Walking Ability was observed. WOMAC score has been used only by *Alberio R *et al*. *[36] at 2 years follow-up, resulting in a mean value of 4.94 (SD ± 9.12) in patients treated with a DMC-THA meaning a good clinical outcome for the patients included in the study (Table 3).

### Complications

Other surgical related complications, excluded Dislocation, were: periprosthetic fracture, Sciatic nerve palsy, Infection, Neck impingement. As expected, periprosthetic fractures and infections resulted to be the most represented. 5 studies out of 12 which were included in the review, reported zero rate of postoperative complications. The rate of implant revision was not considered in this review because of the lack of dates in the studies considered. However, in conclusion, the mean complication rate among the studies was 6% with a range from 23 to 0% (Table 3).

## Discussion

This review was designed to analyze the State of the art of THA with dual mobility articulation outcomes in displaced Femoral Neck Fractures and Hip Osteoarthritis of patients affected by neuromuscular disease (Table 4).

**Table 4:** Dislocation Rates, Complications, and Outcomes

**Ref**	N°of patients	Osteoarticular Diagnose	Approach	Dislocation rate	Complications	Outcomes
*El-Deeb MA *et al*.*[27]	20	Femoral neck fracture	Lateral approach	0	Periprosthetic fracture (1), Sciatic nerve palsy (1), Infection (1)	Post -op Harris Hip Score 92–93
*Liang C *et al*. *[28]	17	Femoral neck fracture	N/A	0	0	n/a
*Ryu HG *et al*. *[29]	35	Femoral neck fracture	Anterolateral (15); Posterolateral (20)	2	Periprosthetic fracture (5), Infection (1)	Post -op Harris Hip Score 81.5 ± 13.5; UCLA Activity score Pre 4.6 ± 1.5;Post 4.6 ± 1.5
*Lazennec JY *et al*. *[3]	42	Hip Osteoarthritis	Anterolateral	0	Periprosthetic fracture (4; 2 cerclage, 2 ORIF), Infection (2)	n/a
*Ochi H *et al*. *[30]	33	Femoral neck fracture	Anterolateral	0	1 not specified complication	Walking ability preop: stage 1 in 28 pz, stage 2 in 1 pz, stage 3 in 1 pz, stage 4 in 0 pz; Walking ability post: stage 1 in 21 pz, stage 2 in 2 pz, stage 3 in 1 pz, stage 4 in 2 pz
*Harwin SF *et al*. *[31]	249	Femoral neck fracture	N/A	0	Neck impingement (1), Infection (1)	n/a
*Iorio *et al*. *[32]	30	Femoral neck fracture	Direct lateral	0	0	n/a
*Nonne D *et al*. *[33]	60	Femoral neck fracture	Posterolateral	0	0	n/a
*Bassiony *et al*. *[34]	13	Femoral neck fracture	Direct lateral	0	Periprosthetic fracture (2), Infection (1)	n/a
*Godoy-Monzon D *et al*. *[35]	41	Femoral neck fracture	Posterolateral	0	0	n/a
*Alberio R *et al*. *[36]	28	Femoral neck fracture	Posterolateral	0	0	Post-op WOMAC 4.94 (SD ± 9.12)
*Graversen AE *et al*. *[18]	20	Femoral neck fracture	Posterolateral	0	0	n/a

Many patients undergoing total hip arthroplasty (THA) are usually affected by FNF. Individuals with FNF are at a higher risk of prosthetic dislocation compared to those with hip osteoarthritis, primarily due to a combination of muscle weakness, cognitive and neurological issues, and frequent falls, which are common characteristics in this patient group [20]. Conditions that directly lead to muscle weakness, especially in the abductors, can increase the likelihood of dislocations. Other neurological disorders, like cerebral palsy and multiple sclerosis, may also cause comparable muscle weakness. In these cases, failure to adhere to or an inability to follow postoperative activity restrictions is often considered the primary factor contributing to dislocations [19].

Our Review suggests that Dual Mobility cups are effective in reducing the risk of dislocation in patients with femoral neck fractures (FNF) and Hip Osteoarthritis (OA) who also suffer from neuromuscular disorders and cognitive impairments, such as those caused by old strokes, polio, intracranial hemorrhage, advanced dementia (including Alzheimer’s), Parkinson’s disease, psychiatric depression, and epilepsy.

This idea is supported by the results we obtained from the studies considered. Infact, general dislocation risk in primary THA in the last decade is 1.7%, with different rates between patients diagnosed with OA and FNF [21]; same implants, in the neurologically impaired population, have an higher risk of complications, with a dislocation rate observed that rates up to 10.6% [22]. Considering the intraprosthetic dislocation of dual-mobility total hip arthroplasty implants, data show an incidence ranging from 0 to 0.3% [23, 24]. The data collected in our systematic review included only the population affected by neurological disorders, and the dislocation rate was still found to be 0.34%, therefore comparable with the observed range of dislocation for dual-mobility cups in the general patient group. In conclusion the implant of choice in the neurologically impaired population in need of a THA appears to be a Dual Mobility cup with a reduction of the risk of dislocation higher than 31 times if compared with standard implants.

In support of this thesis, is relevant to cite *Cnudde PHJ *et al. [37]*.*, who, in 2022, conducted a longitudinal cohort study including 9638 patients with a neurological disease presenting with a femoral neck fracture and treated with hemiarthroplasty (HA), a conventional THA, or a dual-mobility component THA. The one-year dislocation rate they registered was 3.7% after HA, 8.8% after cTHA (with head diameter < 32 mm), 5.9% after cTHA (with head diameter = 32 mm), and 2.7% after DMC-THA. This conclusion means that patients with a neurological disease who are not eligible for THA and should undergo HA, whereas those eligible for THA could benefit a DMC-THA.

All the studies included in this research have shown promising results regarding treatment with DMC THA in patients with neurological disorders not only for what regards lowering the dislocation rate but also for the ability to restore kinetic capabilities. The data collected for this systematic review revealed that treatment for hip osteoarthritis and femoral neck fractures with Dual Mobility Cup THA not only reduces the rate of dislocation, but allows good clinical outcomes and return to a standard of performances in usual life activities comparable with the one had before hip impairment; as reported by *Ochi H *et al. [30]*.* In addition, *El-Deeb MA *et al*.* [27]*, Ryu HG *et al*. *[29] and *Harwin SF *et al*. *[31]*,* using the HHS, registered postoperative gradings between the range of “Good” and “Excellent” in all patients included in their studies.

Future studies should include an in-depth analysis about correlation between DMC-THA dislocation rate in patients with neurological diseases and surgical approach, to verify if the rate remains stable in this specific population as well as general patients.

Further research should focus on the cost-effectiveness of DMC-THA in the neurological impaired population. Dual mobility bearings were created to help lower the risk of dislocation in primary total hip arthroplasty (THA) however, given the significantly higher cost of these implants, a careful balance must be found between the clinical advantage of reducing dislocation risk and the potential for future revisions, alongside the associated economic considerations. Obviously the presence of 2 types of articulations in the DMC have raised concerns about the potential for polyethylene wear and aseptic loosening and consequent necessity of revision surgery. However, recent research has indicated that wear rates between DMC and standard cups are similar [17]. In any case, for elderly patients with neurological conditions and low activity levels, the priority may be a pain-free total hip arthroplasty (THA) that carries a lower risk of dislocation and the need for reoperation, rather than focusing on the potential for polyethylene wear [18].

At the moment, recent studies report that in general population aged > 75 years, dual mobility bearings become cost-saving after 7 years [25] and that DM systems are not cost-effective when the cost of the implant surpassed that of the traditional system by a certain amount of money ( $1023 in reference to US Sanitary System) [26]. So, it would be useful to conduct an economic analysis in this specific population with a higher risk of dislocation and thus revision surgery, in order to confirm a potential cost saving from the implantation of DMC-THA in neurological impaired patient cohort.

The main limitation of this and many other studies on the subject, appears to be the restricted period of follow-up that rarely goes over 5 years, the loss of patients at follow-up due to elder age and the lack of functional outcomes as well as patients reported outcome measures due to the absence in the majority of the studies we screened.

## Conclusion

Results from this study highlight that THA with DMC may be a better solution than standard THA in patients with femoral neck fractures or hip osteoarthritis who experience muscle weakness due to neurological conditions and cognitive impairments due to the possibility of dual mobility cup to prevent early dislocation. Therefore, future studies should also include hip revision surgery and have longer-term follow-up to determine the true benefit of modular dual-mobility articulations in neurological patients.

## Data Availability

No datasets were generated or analysed during the current study.
